# Pseudomyogenic Hemangioendothelioma: A Case of a Solitary Lesion With a Very Indolent Clinical Course

**DOI:** 10.7759/cureus.33172

**Published:** 2022-12-31

**Authors:** Tayler Gant, Chau M Bui, Earl Brien, Bonnie Balzer

**Affiliations:** 1 Pathology and Laboratory Medicine, Cedars-Sinai Medical Center, Los Angeles, USA; 2 Orthopedic Surgery, Cedars-Sinai Medical Center, Los Angeles, USA

**Keywords:** solitary lesion, pseudomyogenic hemangioendothelioma, indolent, vascular, sarcoma, epithelioid, hemangioendothelioma

## Abstract

Pseudomyogenic hemangioendothelioma (PMH), also known as epithelioid sarcoma-like hemangioendothelioma, is a rare epithelioid vascular neoplasm predominantly affecting young adult males at an average age of approximately 30 years. This tumor is rare; therefore, detailed information regarding this tumor is still lacking.

Here, we report a case of a man in his 20s presenting with left foot pain for about one year. Imaging showed a 2-cm ovoid, cortically based lesion with a lytic defect of the cortex at the fifth metatarsal proximal shaft. Histologically, the lesion presented as an infiltrating proliferation of distinctly myoid-appearing spindled cells with eosinophilic cytoplasm and mildly atypical vesicular nuclei. Scant mitoses were identified with no areas of necrosis. Tumor cells exhibited strong, diffuse cytokeratin expression as well as CD31 and ERG. CD34 was positive in a few tumor cells, and integrase interactor 1 (INI1) retained nuclear expression. No reactivity for S100, desmin, smooth muscle actin (SMA), epithelial membrane antigen (EMA), and CD1a was present.

Over half of the patients with PMH develop multifocal lesions, often involving several tissue planes; however, distant metastasis is very infrequent. This patient underwent curettage and internal fixation of the left fifth metatarsal and had no evidence of recurrence or distant metastasis after seven years of follow-up. Our case contributes to the growing knowledge of PMH and sheds light on the prognosis of these lesions.

## Introduction

Pseudomyogenic hemangioendothelioma (PMH) is an endothelial neoplasm, mostly indolent and with low-grade malignancy, having several different classifications. The current nomenclature was originally described by Hornick and Fletcher (2011), in which its myoid and epithelioid-like histologic pattern is defined as "pseudomyogenic" [1]. It is predominantly found in young adult males, at approximately 30 years of age, and in the distal extremities [1]. This vascular neoplasm is multifocal and can involve the dermis, subcutis, and bone [2]. The multifocality of these tumors tends to involve the same local anatomical regions as the original tumor [3]. This tumor is rare; therefore, investigation for additional detailed information regarding this tumor is warranted.

## Case presentation

**Figure 1:**
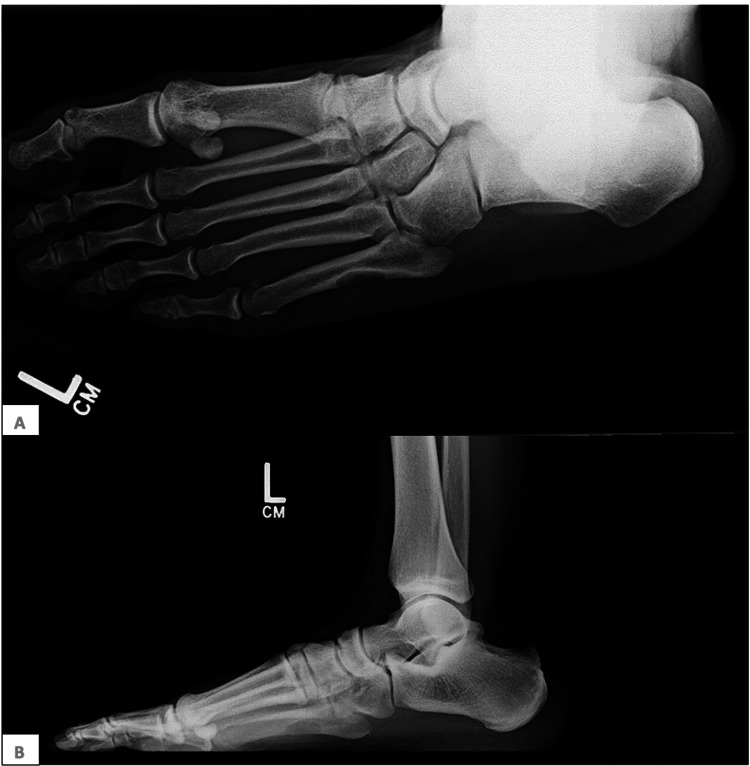
Pseudomyogenic hemangioendothelioma X-rays of ovoid, cortically based lesion at the proximal fifth metatarsal shaft. (A) Anterior view; (B) lateral view.

A male in his 20s presented with increasing pain in his left foot for the past year. He had a history of hypertension but had no other medical problems or trauma. A physical exam revealed tenderness over the fifth metatarsal, particularly at the proximal shaft. There was no warmth, erythema, or lymphadenopathy, and a reassuring distal neurovascular exam. Plain X-rays revealed a 2-cm ovoid lytic lesion in the fifth metatarsal, with a saucer-like deformity and significant scalloping, favoring reparative granuloma, chondroma, periosteal desmoid, or other neoplasms (Figure 1). The lesion was also identified on CT and MRI. A biopsy of the lesion was taken at the time of surgery.

**Figure 2:**
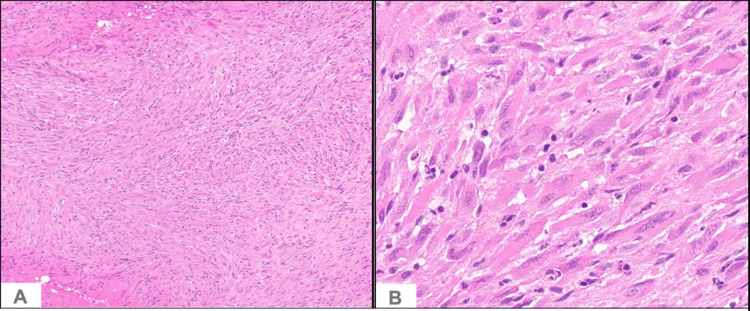
Pseudomyogenic hemangioendothelioma Infiltrating proliferation of distinctly mysid-appearing spindled cells with eosinophils cytoplasm, middle atypical vesicular nuclei, and scant mitosis. (A) Hematoxylin and eosin (H&E), 100x magnification; (B) H&E, 400x magnification.

Histopathological examination of the biopsied specimen revealed infiltrating proliferation of distinctly myoid-appearing spindled cells with eosinophilic cytoplasm and mildly atypical vesicular nuclei (Figure 2).

**Figure 3:**
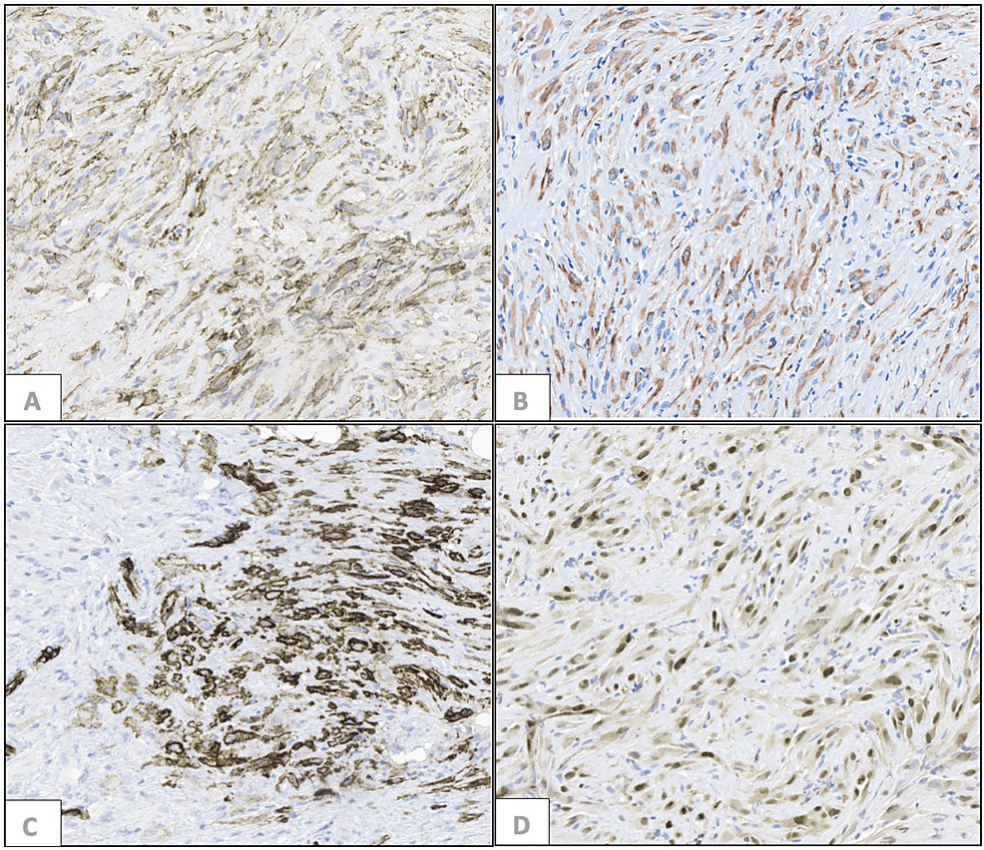
Pseudomyogenic hemangioendothelioma (A) Diffusely positive AE1&3 stain. (B) Positive CD34 stain in a few tumor cells. (C) Diffusely positive CD31 stain. (D) Diffusely positive ERG stain (400x magnification).

Scant mitoses were identified with no areas of necrosis. Tumor cells exhibited strong, diffuse cytokeratin AE1&3 expression, as well as CD31 and ERG (Figure 3). CD34 was positive in a few tumor cells (Figure 3), and integrase interactor 1 (INI1) retained nuclear expression. No reactivity for S100, desmin, smooth muscle actin (SMA), epithelial membrane antigen (EMA), and CD1a was present.

Next-generation sequencing (NGS) panel with 58 gene fusions (Cleveland Clinic Foundation panel) was performed and no gene fusion was detected. These findings are consistent with an epithelioid sarcoma-like epithelioid hemangioendothelioma (EHE), with a differential diagnosis including epithelioid vascular neoplasm and epithelioid sarcoma. The retention of INI1 expression, strong CD31 positivity, and lack of diffuse CD34 argue against epithelioid sarcoma, which was described as PMH.

**Figure 4:**
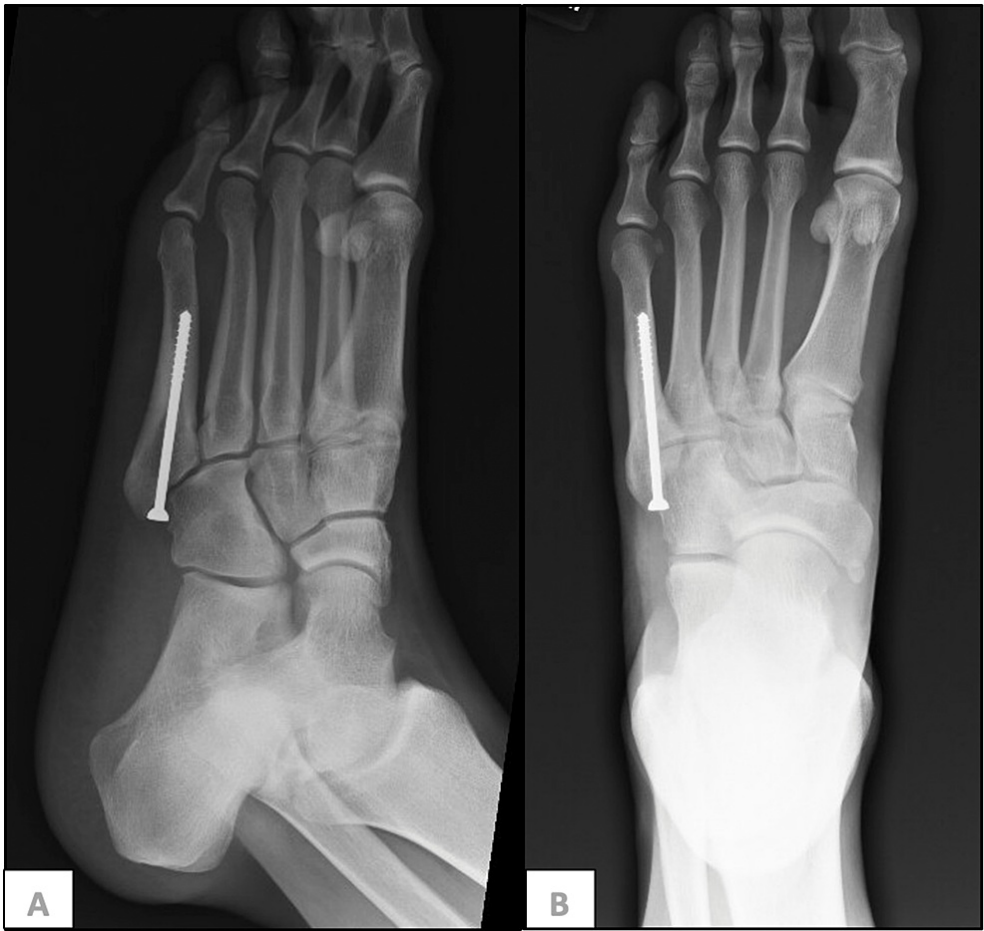
Post-surgical X-rays X-rays of stable screw fixation and post-surgical changes of the fifth metatarsal, without radiographic evidence of recurrent tumor or hardware complications. (A) Anterolateral view; (B) anterior view.

The decision was made to treat the lesion with debridement, curettage, grafting, and internal fixation. Post-surgical follow-up positron emission tomography (PET) scans were negative, and CT scans have shown progressive healing of the pathologic fracture and the cavitating cortical lesion (Figure 4). There was no evidence of recurrent tumor, including local recurrence or local spread to other sites after seven years of follow-up.

## Discussion

PMH is a rare tumor, mainly occurring in males with an average age of 31 years [1]. PMH can be asymptomatic, but the most common symptom is pain in the region of the tumor [4]. It most commonly occurs in the dermis and subcutis tissue layer, yet approximately half of the cases have intramuscular lesions, and 20% are intraosseous [1]. Grossly, 70% of these lesions are multifocal, unlike in this case as a solitary lesion, and average in size between 1 and 2.5 cm [2,5]. It is a mostly indolent tumor with a low likelihood to metastasize, as also demonstrated in this case [1]. The main treatment for PMH is debridement; however, chemotherapy is also used. Targeted angiogenic drugs have been proposed, specifically as a treatment for vascular tumors. Sirolimus is a mammalian target of rapamycin (mTOR) pathway inhibitor drug in which mTOR is prevented from activating protein synthesis and angiogenesis. Sirolimus (rapamycin) and derivatives have been predicted to be an effective and less toxic treatment compared to chemotherapy for PMH [2].

Histologic morphology of PMH consists of neoplastic cells that are enlarged, spindled, and with bright eosinophilic cytoplasm that can mimic rhabdomyoblasts. They contain mild nuclear atypia and infrequent mitotic activity [1,6].

PMH has an inclusive differential diagnosis of epithelioid sarcoma, rhabdomyosarcoma, osteoblastoma, and vascular tumors [4]. PMH has presenting similarities with epithelioid sarcomas, such as presenting in the skin and soft tissue in the distal extremities and having diffuse keratin positivity [1]. This case represents the importance of identifying and distinguishing PMH from other vascular tumors with poorer prognoses. To differentiate between PMH and epithelioid sarcoma, as both have cytokeratin positivity, epithelioid sarcoma expresses MNF116 and AE1&3 and has a loss of expression of INI1 (90%) and CD34 (50%) [3,7]. PMH is also negative for pan-cytokeratin, EMA, and CD34, in which 50% are positive in epithelioid sarcoma [1,3,7]. Histologically, PMH can mimic other skeletal muscle tumors; however, PMH lacks expression for actin, MyoD1, and desmin [1,6,8]. PMH usually shows reactivity with vascular markers, including CD31, FLI1, and ERG, as well as co-expresses vimentin and keratins such as AE1&3, CK7, and CAM5.2 [6].

Another useful tool in the diagnosis of PMH is the use of FOSB immunohistochemistry. The FOSB expression for PMH is strongly positive [9]. The SERPINE1-FOSB gene fusion due to the chromosomal translocation t(7;19)(q22;q13) has been identified in several cases [6,10]. The WHO 2020 classification reported on recently identified genetic alterations in which PMH has SERPINE1-FOSB and ACTB-FOSB, which can be useful when distinguishing PMH from other vascular tumors [10]. The differential diagnosis for PMH can be broad, and the rarity emphasizes the importance of immunohistochemical stains and increases the possibility of being overlooked.

## Conclusions

PMH is a rare neoplasm, particularly with the involvement of the bone. It is characterized as a multifocal, male-predominant tumor with predominantly spindled epithelioid morphology and positive keratin markers. We presented a patient who underwent curettage and internal fixation of the left fifth metatarsal and had no evidence of recurrence or distant metastasis after seven years of follow-up. Our case contributes to the growing knowledge of PMH and sheds light on the importance of the identification of these lesions and their effects on treatment and prognosis.
